# Optimisation of the WC-Co Composite Manufacturing Process Using Spark Plasma Sintering Technology with the DOE Methodology

**DOI:** 10.3390/ma19071278

**Published:** 2026-03-24

**Authors:** Robert Kruzel, Tomasz Dembiczak, Zbigniew Bałaga, Marcin Lis, Dariusz Kołacz, Joanna Wachowicz, Sylvia Kuśmierczak, Nataša Náprstková

**Affiliations:** 1 Faculty of Civil Engineering, Czestochowa University of Technology, Akademicka Street 3, 42-201 Czestochowa, Poland; 2 Faculty of Science and Technology, Jan Dlugosz University in Czestochowa, Armii Krajowej Street 13/15, 42-200 Czestochowa, Poland; 3 Faculty of Production Engineering and Materials Technology, Czestochowa University of Technology, Armii Krajowej Street 19, 42-201 Czestochowa, Poland; zbigniew.balaga@pcz.pl; 4 Centre of Powder and Composite Materials, Łukasiewicz Research Network—Institute of Non-Ferrous Metals, Sowińskiego 5 Street, 44-100 Gliwice, Poland; marcin.lis@imn.lukasiewicz.gov.pl; 5 Centre of Advanced Materials Technologies, Łukasiewicz Research Network—Institute of Non-Ferrous Metals, Sowińskiego 5 Street, 44-100 Gliwice, Poland; dariusz.kolacz@imn.lukasiewicz.gov.pl; 6 Institute of Wood Sciences and Furniture, Department of Mechanical Processing of Wood, Warsaw University of Life Sciences, Nowoursynowska Street 166, 02-787 Warsaw, Poland; joanna_wachowicz@sggw.edu.pl; 7 Faculty of Mechanical Engineering, Jan Evangelista Purkyně University in Ústí nad Labem, Pasteurova Street 1, 400 96 Ústí nad Labem, Czech Republic; sylvia.kusmierczak@ujep.cz (S.K.); natasa.naprstkova@ujep.cz (N.N.)

**Keywords:** design of experiment (DOE), spark plasma sintering (SPS), cemented carbides

## Abstract

The research conducted in this paper is a practical example of the Design of Experiments methodology. In accordance with the assumptions of the experimental design, the authors drew attention to the problem: how should the spark plasma sintering process be planned to obtain the maximum amount of information needed to optimise the consolidation of the WC-6Co composite at the lowest possible cost? The DOE methodology—a powerful technique for investigating new processes and gaining knowledge about existing ones in order to optimise them for high performance—was employed in the study. The aim of the research was to optimise the consolidation of the spark-plasma sintering process of the WC-6Co composite using the DoE (Design of Experiments) methodology. Four sintering factors were selected for the study: sintering temperature (factor A, 1300–1400 °C); heating rate (factor B, 100–300 °C/min); sintering time (factor C, 150–600 s); and pressure (factor D, 40–50 MPa). Each consolidation factor was designed to cover three levels. The L9 orthogonal array was used. It was found that sintering temperature and heating rate had the greatest impact on apparent density. To validate the statistical model, sintering tests were performed at a temperature of 1380 °C, a heating rate of 100 °C/min, a sintering time of 150 s and a pressing pressure of 45 MPa. Validation analysis of the statistical model demonstrated consistency with the experimental results. The WC-6Co composite achieved an apparent density of 14.85 g/cm^3^, corresponding to 97.42% of the theoretical density, with a hardness of 1809 HV30 and total porosity of 2.583%. X-ray diffraction studies revealed the presence of tungsten carbide and cobalt in the structure.

## 1. Introduction

The smart industry is an interconnected system of machines, communication mechanisms and computing power that utilises advanced technologies.

To achieve faster, more accurate and more affordable production while ensuring high-quality final products, new advanced solutions are required from all components involved in production processes. Cutting tools are no exception; they must be able to cope with the most challenging materials while offering the longest possible service life. The tools used in production processes significantly impact the final machining result, which is why the tool industry is an extremely important sector for the entire industrial processing market, alongside the machinery industry. The appearance of new solutions and technologies on the market, as well as the increasing demands of various industries, forces tool manufacturers to continually adapt their product ranges to new realities. In short, progress in many sectors of the economy would be impossible without new solutions in tool offerings [1,2].

For manufacturing companies, ensuring high competitiveness and product quality is essential for achieving repeatable and stable core production processes. A properly designed and implemented process creates the potential to achieve the intended results, and controlling its individual components and raising employee awareness of their importance are key to optimising it. Continuous improvement increases the efficiency of processes and impacts the functioning of the entire enterprise management system [3,4,5]. Powder metallurgy is a dynamic technology with versatile applications in many industries, and its scope is gradually expanding [6].

### 1.1. Spark Plasma Sintering (SPS)

Powder metallurgy is a technology used to produce sintered materials from metallic or non-metallic powders. The process involves producing powders or powder mixtures of various materials, forming shapes, sintering them and finishing the sintered material. Sintering is a thermally activated mass transfer process in which loosely bound powder particles consolidate into a solid material through diffusion. This process leads to further densification of the sinter [6]. Sintering is the most important step in the powder metallurgy process because it directly affects the strength, dimensional accuracy, shape and functional properties of the final product [6]. Depending on the sintering conditions, a distinction is made between free sintering and pressure sintering.

**Figure 1:**
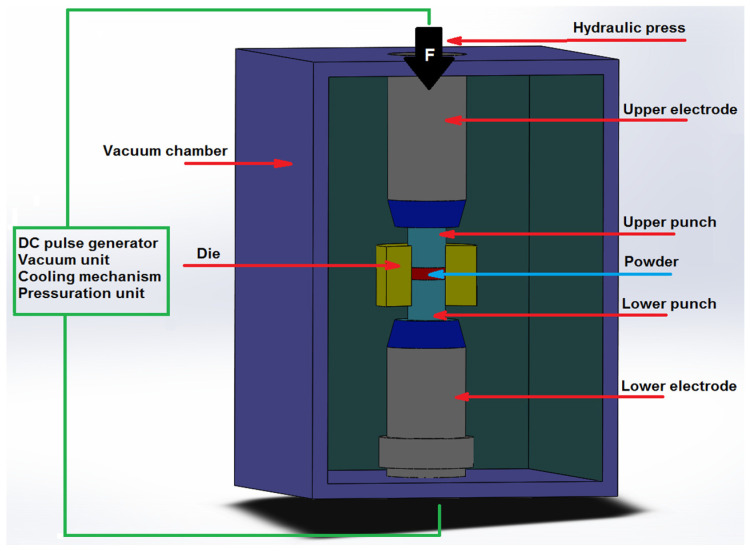
Schematic diagram of a Spark Plasma Sintering device. Developed on the base [10].

Many powder sintering methods were developed over the 20th century. One of these is spark plasma sintering (SPS). This method enables a wide range of materials to be sintered at lower temperatures and pressures than with free sintering. SPS enables materials to be shaped without the need for pre-pressing, isostatic densification or drying [7,8,9,10]. Figure 1 shows a schematic of an SPS device.

Over the years, numerous scientific publications have been produced in the field of powder metallurgy, using the Spark Plasma Sintering method to produce sintered materials from various substances.

In their scientific publication on Spark Plasma Sintering (SPS) technology, Suarez et al. [11] describe SPS as a rapid sintering technique in which heat is distributed uniformly and rapidly throughout the powder mass. The heat is supplied by a low-voltage pulsed direct electric current, while a low-pressure uniaxial piston applies a force to consolidate the powder materials. The generated Joule heat is delivered directly to the powder. Using high sintering temperatures, pressures and heating rates promotes rapid sintering processes and the production of high-density materials. These materials possess excellent mechanical, thermal and chemical properties with minimal microstructural defects. Cha et al. [12] demonstrated that 100 nm WC powder could be sintered at 1000 °C with a heating rate of 100 °C/min using SPS technology and that ultrafine particles measuring approximately 300 nm could be produced without the addition of an inhibitor.

The authors of [13] conducted research aimed at obtaining the intermetallic compounds Bi_2_Te_3_, Bi_0.4_Sb_1.6_Te_3_ and Bi_2_Te_2.95_Se_0.05_ using a method combining mechanical synthesis and spark plasma sintering. Based on their research, the authors concluded that mechanical alloying alone enables the production of pure Bi_2_Te_3_ and Bi_2_Te_2.95_Se_0.05_ compounds. In the Bi_0.4_Sb_1.6_Te_3_ alloy, only plasma sintering causes the alloyed metals to react completely. Consolidation of the obtained powders by plasma sintering under specific conditions allows them to retain their nanocrystalline structure obtained during mechanical alloying.

The authors of [14] presented the results of structural studies, hardness and microhardness measurements, and compressive strength tests of the AK52/SiC composite obtained by powder metallurgy methods. The starting material was produced from AK52 (AlSi_5_Cu_2_) alloy chips with the addition of silicon carbide (SiC) in a mechanical synthesis process. A fine-grained powder with a uniform distribution of hard ceramic particles in an aluminium matrix was obtained. The consolidation process was carried out using the pulse plasma sintering method, which allowed mouldings with good mechanical properties and a homogeneous structure to be obtained in a short time. The addition of hard SiC particles resulted in an increase in the hardness and strength of the samples produced. Structural analysis confirmed the uniform structure and fine grain size of the mouldings obtained.

The authors of [15] investigated the possibility of direct synthesis of Zn_4_Sb_3_ using spark plasma sintering and analysed the effect of synthesis parameters on material properties. Materials with different initial compositions were sintered into multiphase samples consisting of a Zn_4_Sb_3_ matrix containing ZnSb, Zn and Sb inclusions. For each initial stoichiometry, the sintering temperature and time were limited in different ways by the distribution of the desired β-Zn_4_Sb_3_ phase. The authors demonstrated that high concentrations of inclusions deteriorate the thermoelectric properties of Zn–Sb-type materials. In a material with a stoichiometric initial composition, a high power coefficient of 1200 μW/(m K^2^) was found at 580 K. An interesting correlation was discovered between the improvement of thermoelectric properties and the depletion of the β-Zn_4_Sb_3_ phase in zinc.

The authors of [16] presented the results of their work on the technology of producing W-Re, Mo-Re and TiO_2_ targets with tungsten, neodymium and hafnium, using a modern powder metallurgy technique known as plasma spray sintering (SPS). The method used allowed for the production of targets with high density and low porosity. Tests on the production of thin layers using the magnetron method with the targets obtained confirmed that they can be used for magnetron sputtering.

The authors of [17] presented the results of research on silver-based nanocomposite materials with the addition of multi-walled carbon nanotubes. Powder with a carbon nanotube content of 0.1 to 3% by weight was produced using powder metallurgy methods, through mixing and high-energy grinding, as well as chemical methods. The modification of carbon nanotubes involved the electroless deposition of silver particles on the active surfaces of carbon nanotubes and chemical reduction with a strong reducing agent—sodium borohydride (NaBH_4_). The resulting powder mixtures were consolidated using the SPS (Spark Plasma Sintering) method. The resulting composites were tested for relative density, electrical conductivity and electroerosion properties. The tests also included a detailed analysis of the structure using X-ray microanalysis, taking into account the distribution of carbon nanotubes. The influence of the manufacturing methods on the properties of the obtained composites was observed.

The authors of [18] presented the results of tests on W-TiB_2_ composite sintered materials used as electrodes in the electro-spark deposition (ESD) process and tests on the deposited layers. The scope of the research included a detailed characterisation of powder mixtures, composite sintered materials produced by spark plasma sintering (SPS) and layers deposited by electro-spark deposition. The ESD process, using a W+30% vol. TiB_2_ electrode, was carried out using an automated device. The substrates were made of copper and aluminium. The paper also presents a topographical analysis of the composite layer surface and an assessment of its wear resistance. The analysis of the test results showed that it is possible to obtain, using the SPS method, good quality composite materials that can be used as electrodes in the electro-spark process. The obtained layers were characterised by increased wear resistance compared to the substrate material.

The authors of [19] produced Cu-graphite composites by plasma sintering of copper and graphite powders in the range of 15–80% vol. of graphite in order to analyse the dependence of the coefficient of friction and wear rate on the composition of Cu-graphite composites. The authors observed that with increasing graphite concentration, the coefficient of friction and wear rate of the composites initially decrease from the values of copper. After reaching a critical graphite concentration threshold (below 15% graphite by volume), the friction coefficient of the composites becomes independent of the composition, reaching values in the range of 0.15–0.16 relative to a 100Cr6 steel mandrel. Apart from the fact that there are similar studies of Cu-graphite composites, none of them examined the Cu-graphite system prepared in the widest possible range of compositions with different technologies and the same powders. It was observed that the dependence of the friction coefficient on the composition does not depend on the different preparation methods (hot isostatic pressing, spark plasma sintering) for identical copper and graphite powders. The wear rate of composites increases from 2.2 × 10^−4^ mm^3^ N^−1^ m^−1^ at 15 vol. % graphite to 3.8 × 10^−3^ mm^3^ N^−1^ m^−1^ at 80 vol. % graphite. At low graphite content, adhesive wear occurs: the wear path is U-shaped with raised edges. At high graphite concentrations, abrasive wear occurs with a wide, straight U-shaped wear path. The microhardness of the tested composites decreases linearly from 62 MHV for copper to 2.7 MHV for graphite.

### 1.2. WC-Co Composites for Cutting Tools

The production of parts and tools using powder metallurgy technology is currently on a par with metal forming technologies such as forging and casting. The competitiveness of powder metallurgy production stems from the high quality of parts for many important applications, as well as advantages in terms of material utilisation, shape complexity and dimensional control close to the net shape. These factors contribute to sustainable development, establishing powder metallurgy as a green technology [20].

The basic tool material used in modern industry is cemented carbide. It is a multi-component material in which carbide particles are bonded using a binder. The carbide content ranges from 70 to 97%, and the grain size from 0.4 to 25 μm. Cemented carbide’s basic structure consists of tungsten carbide (WC) and cobalt (Co), which form different carbide grades depending on the percentage of each element present. Tungsten carbide forms the hard layer, while cobalt acts as a binder. As well as the aforementioned combinations of tungsten carbide and cobalt, other combinations use titanium carbide (TiC), tantalum carbide (TaC) and niobium carbide (NbC), as well as iron (Fe), chromium (Cr), nickel (Ni) and molybdenum (Mo) alloys [21]. Cemented carbides produced using powder metallurgy technology have diverse performance characteristics that can be tailored to the materials being processed. The properties of cemented carbides primarily depend on the content of the binder phase, the size of the powder particles, how the mixture is prepared, how it is pressed, and the sintering parameters [21].

**Figure 2:**
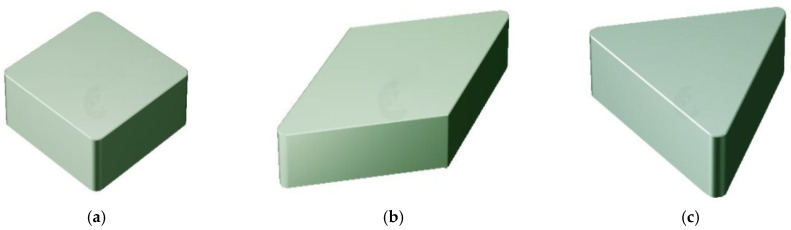
Examples of carbide cutting edges used in machining: (**a**) square insert, (**b**) rhombic insert, (**c**) triangular insert. Developed on the base [22].

Cemented carbide tools are characterised by their high hardness and abrasion resistance. They can also withstand very high temperatures and are used for machining hard materials. They are used in virtually all machining processes, including milling, turning, drilling, boring and threading [22]. Figure 2 shows examples of cutting edges manufactured by powder metallurgy for use in machining.

### 1.3. Design of Experiments (DOE) in Materials Processing

The quality of products manufactured using powder metallurgy technology depends on the parameters of the mixing, pressing and sintering operations (i.e., heating rate, sintering temperature and time, and pressing pressure), as well as the protective atmosphere. The Design of Experiments (DoE) methodology is essential for selecting the optimal manufacturing process conditions. DoE is a key method in engineering and industry, enabling the systematic study of the effects of various factors on processes. DoE is an effective analytical tool that helps engineers to better understand processes and phenomena by enabling them to plan and conduct experiments efficiently and purposefully. The lessons learned from DoE are applicable not only in scientific research but also in industry, where they can be used to optimise production processes, improve products, enhance quality, reduce variability and minimise costs. The design of experiments methodology enables more informed decisions to be made based on solid empirical data, thus contributing to progress in various fields of science and technology [23,24].

The aim of planning an experiment is to answer a question. How should an experiment be designed to obtain the most useful information at the lowest possible cost? In practice, this involves creating a mathematical model that describes the behaviour of the process being studied. The outcome of this process is the identification of input variables that significantly impact the controlled process. Identifying the relationship between input parameters and the process guarantees the optimal result, i.e., the maximum number of defect-free products possible:

– an optimal result, i.e., the maximum possible number of defect-free products,
– minimal process variability;
– minimal sensitivity of the process to uncontrollable changes.

From a mathematical perspective, experiments determine a function representing the relationship between the factors influencing the studied process and its results (outputs) [23,24].

In their study, Ujah et al. [25] optimised the sintering parameters (i.e., sintering temperature, heating rate, pressing pressure and sintering time) for a sintered Al-CNTs-Nb nanocomposite using the Taguchi experimental design. There was close agreement between the DoE results obtained using the Taguchi method and the experimental results (density and hardness). The Taguchi method is a statistical DOE model that can be used for process optimisation. Mohammadadeh et al. [26] worked on optimising the physical and mechanical properties of a titanium-titanium diboride composite. They employed the response surface methodology (RSM) DoE. The experiments were conducted based on a factorial design derived from the RSM report. It was found that sintering temperature had the greatest influence on the properties of density, porosity and hardness.

The authors in [27,28] applied the design of experiments (DoE) methodology to improve the quality of laser cutting of 10 mm thick S355J2C+N steel. They used response surface methodology (RSM) to develop a model of the laser cutting process. The results showed that the surface roughness value is significantly affected by the cutting speed, the interaction between speed and peak power, the interaction between focus and speed, and the peak power parameter.

The authors in [29] optimised the isothermal quenching of cast iron with compacted graphite (CGI). They used the response surface method (RSM) for optimisation. The results obtained were compared with the proposed mathematical models. Based on ANOVO analysis, they concluded that the isothermal quenching temperature (Tpi) had the greatest impact on each parameter studied. The optimal conditions for the analysed parameters, assuming maximum tensile strength and yield strength and elongation of approximately 0.7%, were obtained for the following heat treatment parameters: Tγ = 890 °C; Tpi = 290 °C; τγ = 120 min; τpi = 150 min.

### 1.4. Innovation and Scope of Work

The aim of the research was to optimise the parameters of the sintering process in Spark Plasma Sintering technology of WC-Co composites using the Genichi Taguchi method.

The influence of four sintering parameters was examined: heating rate, sintering time, sintering temperature and pressing pressure.

An innovative element of the work is the experimental design, which was carried out on the basis of orthogonal arrays, which allowed the influence of many variable sintering parameters to be investigated with a minimum number of trials at the very beginning. This innovative approach to research provided new knowledge about the influence of individual parameters of the sintering process in Spark Plasma Sintering technology of WC-Co composites.

The optimisation of sintering parameters contributed to a reduction in the consumption of powders needed to produce samples and a reduction in the costs associated with the operation of the Spark Plasma Sintering equipment.

## 2. Materials and Methods

### 2.1. Material Descriptions

The material used to produce the test samples was WC powder with an average particle size of 1 µm and a purity of 99.5%, as well as cobalt (Co) powder with an average particle size of 1.5 µm and a purity of 99.8%. The powder mixtures were prepared in a Turbula mixer using 10 mm diameter Al_2_O_3_ balls at a ratio of 1:2, resulting in a mixture with a chemical composition of 94% WC and 6% Co (by weight). The mixing time was six hours at a rotational speed of 100 rpm.

### 2.2. Design of the Experiment

DOE is helpful in analysing all possible combinations of process parameters. The impact of various factors causes changes in experiments, which can be determined through experimental design. In Taguchi’s method, the concepts of orthogonal arrays and signal-to-noise ratios (S/N) were adopted for data analysis and prediction of optimal results. The range of sintering factors was selected based on the authors’ experience: sintering temperature (A, 1300–1400 °C), heating rate (B, 100–300 °C/min), sintering time (C, 150–600 s) and pressure (D, 40–50 MPa): sintering temperature (A, 1300–1400 °C), heating rate (B, 100–300 °C/min), sintering time (C, 150–600 s) and pressure (D, 40–50 MPa). For the four sintering factors, each factor is designed with three levels, as shown in Table 1. This is because the influence of these factors on the result may vary nonlinearly. An L9 (34) orthogonal array (OA) was employed, as it was able to provide the minimum degrees of freedom required for the experimental exploration. The column assignment and the experimental layout are shown in Table 2. After completing all experiments and the analysis of the data, a confirmatory experiment was performed at the recommended settings. This is an important step in Taguchi’s parameter design, as it provides indications on the validity of the experimental procedures and results.

### 2.3. Spark Plasma Sintering

The sintering process was carried out using an FCT Systeme Spark Plasma Sintering furnace (Frankenblick, Germany) The sintering out under vacuum was carried out in tools made from graphite. The charging chamber in the graphite tools unit was filled with powder. Graphite foil was placed between the powder, die and punch. The prepared tool was placed in the sintering chamber. Sintering processes were carried out according to the design of the experiment in Table 2.

### 2.4. Density Measurement

The apparent density was measured using the hydrostatic weighing method, which is based on Archimedes’ principle and in accordance with PN-EN ISO 3369:2010 [30]. Measurements were taken using a RADWAG AS 220.R2 PLUS laboratory balance (Radwag, Radom, Poland). Hydrostatic weighing measurements were carried out at a liquid temperature of T = 24 °C. Distilled water was used for density measurements. The apparent density was calculated using the following formula [30]:

(1) $$\rho_{b}=\frac{m_{1}}{m_{3}-m_{2}}\times \rho_{L}$$

where: m_1_—dry sample mass, m_2_ apparent mass of the sample immersed in liquid, m_3_ mass of the sample saturated with liquid, and ρ_L_—liquid density depending on temperature.

The porosity was determined from the relationship [30]:

Open porosity P, expressed as a percentage, is the ratio of the volume of open pores in the sintered material to its external volume. Open porosity was calculated according to the formula:

(2) $$P_{o}=\left(\frac{m_{3}-m_{1}}{m_{3}-m_{2}}\right)\times 100\%$$

Total porosity takes into account both open and closed pores. The mathematical expression is given by the following formula:

(3) $$P_{c}=\left(1-\frac{\rho_{p}}{\rho_{t}}\right)\times 100\%$$

Closed porosity is expressed as a percentage and represents the difference between total porosity and open porosity:

(4) $$P_{z}=\;\left(P_{c}-P_{o}\right)\times 100\%$$

### 2.5. Taguchi Method

The Taguchi method aims to optimise the quality of multi-parameter processes through experimentation. In his research, Genichi Taguchi observed that each product in operation generates a certain amount of loss. This loss is inversely proportional to product quality. According to Taguchi, this justifies using loss as the primary measure of product quality decline. Taguchi’s method can be used to optimise new processes (products) and improve existing ones.

In his method, Taguchi used the terms ‘signal’ and ‘noise’, representing the expected (average) and undesired (standard deviation) values of the response, respectively. Based on response requirements, Taguchi divided the S/N ratio into three categories: ‘average’–the better; ‘higher’–the better; and ‘lower’–the better. In this study, the quality characteristic is apparent density: the higher the apparent density of the sinter, the better its mechanical properties. Therefore, Equation (5) (‘higher’ is better) was used to calculate the S/N ratio, and the results are presented in Table 2. Taguchi analysis was performed using Minitab to obtain graphs of the mean S/N ratios and the results of the analysis of variance (ANOVA). A higher signal-to-noise ratio indicates better performance [31]:

(5) $$S/N=-10{log}_{10}\left(\frac{1}{n}\sum_{i=1}^{n}\frac{1}{y_{i}^{2}}\right)$$

where: n—no. of observations, y—observed data for each response.

### 2.6. Mechanical Properties

Measurements of HV hardness, brittle fracture resistance K_IC_, porosity and X-ray diffraction structure analysis were revealed for a validated model predicting apparent density with optimal sintering parameters.

Vickers hardness measurements at a load of 294.2 N were performed in accordance with using an FM-700 hardness tester (FUTURE-TECH CORP., Kawasaki Ward, Japan) [32].

The critical stress intensity factor KI_c was determined based on the relationship

(6) $$K_{Ic}=0.15\sqrt{\frac{HV30}{\Sigma l}}$$

where HV30 is the hardness measured under a load of 294.2 N and Σl is the sum of the lengths of the cracks formed at the corners of the indentation.

**Figure 3:**
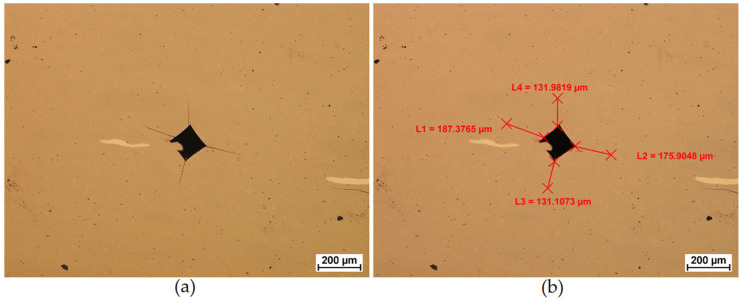
The WC-6Co composite produced with optimal consolidation parameters during Spark Plasma Sintering: (**a**) cracks formed in the corners of the imprint, and (**b**) sum of the lengths of the cracks that have formed in the corners.

Figure 3 shows the method of determining the crack length according to relation 6.

## 3. Results and Discussion

**Figure 4:**
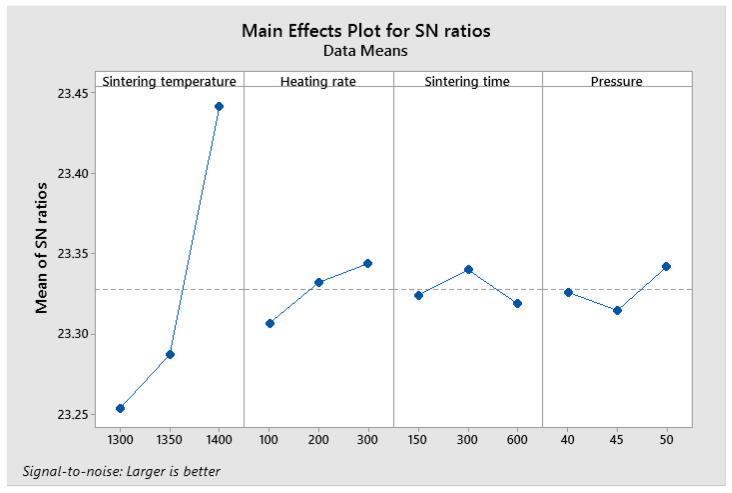
Mean S/N ratio of density.

The obtained S/N ratio response table for density is shown in Table 3; Figure 4 represents the mean S/N ratio graph obtained in the Minitab 19 software tool. A higher S/N ratio represents the minimum variation difference between the desirable output and measured output. From Figure 4, it was noticed that the highest mean S/N ratio obtained for density is sintering temperature at 1400 °C, heating rate 300 °C/min, sintering time 300 s and pressure 50 MPa. This predicted optimum combination was represented as A3-B3-C2-D3.

**Figure 5:**
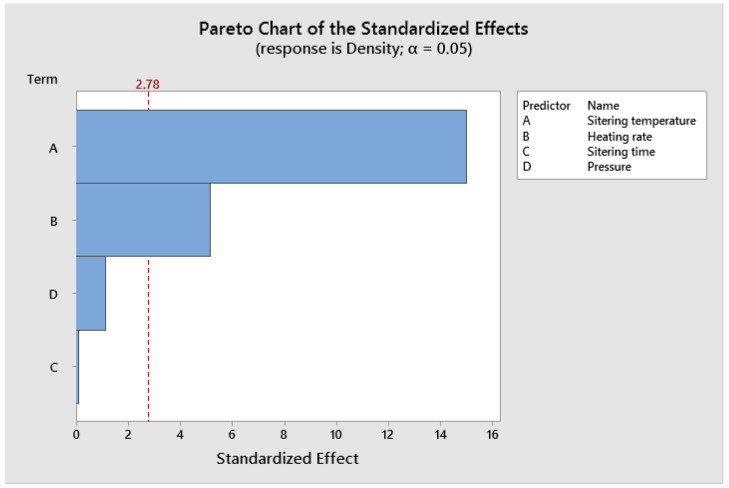
ANOVA results obtained for density.

Analysis of variance (ANOVA) indicates the process parameter that has the greatest influence on performance characteristics. Based on the analysis of variance shown in Figure 5, it was found that density is significantly dependent on sintering temperature, heating rate, pressure and sintering time. The percentage contributions of sintering temperature, heating rate, pressure and sintering time were 93.85%, 3.38%, 1.69% and 1.08%, respectively, as shown in Table 4.

ANOVA analysis shows that the sintering temperature has the greatest impact on the consolidation efficiency of WC-6Co composites using the Spark Plasma Sintering method (Table 4). The main mechanisms of the Spark Plasma Sintering process include pulsed direct current (DC), uniaxial pressing pressure and heating rate [33]. In the initial phase of the process, a high-intensity pulsed current flows through the contacts between the powder particles, which leads to the generation of spark discharges in the pores. These discharges remove oxides and impurities from the surface of the WC and Co particles, activating them, which leads to the particles bonding together. Heat is generated inside the sintered material, causing local melting of the Co (cobalt) binder phase, which contributes to shortening the sintering time. The applied pressing pressure causes plastic deformation of cobalt (Co), which fills the pores between the WC grains. The cobalt (Co) phase undergoes rapid melting and solidification, which is crucial for rapid densification. In this study, in the sintering temperature ranges of 1300–1400 °C, the main mechanism of densification is cobalt (Co) diffusion and grain boundary diffusion, which leads to an increase in the necks between particles. At higher sintering temperatures, the dislocation climbing mechanism is activated in the cobalt (Co) phase, supporting full densification [34].

In the present study, linear regression analysis in the Minitab software tool has been used to develop the mathematical model for the dependent variables of density as a function of sintering temperature, heating rate, sintering time and pressure. The predictive equations obtained from the regression analysis are shown in Equation (7):

(7) $$\begin{array}{cc} Density=10.178 & +\left(0.0032\times Sintering\;temperature\right)\\ & +\left(0.000317\;\times Heating\;rate\right)\\ & -\left(0.000037\;\times Sintering\;time\right)+(0.00267\;\times Pressure) \end{array}$$

**Figure 6:**
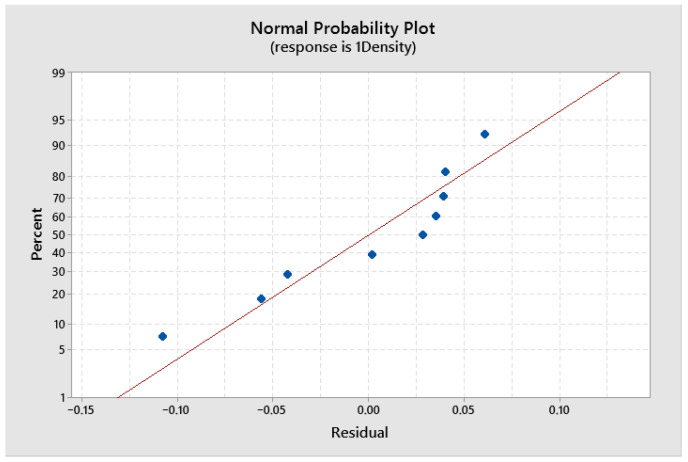
Normal probability plot of the residuals for density.

The capability of the developed model was checked by using a coefficient of determination, R^2^. The coefficient of determination value varies from zero to one. If it is close to one, it means that there is a good fit between the dependent and independent variables. Suppose R^2^ = 95%; then it means that new observations were estimated with 95% variability. In the present study, the developed regression models for density are having high R^2^ values, such as 86.29%. The residual plot was used to check the significance of the coefficients in the predicted model. If the residual plot is a straight line it means that the residual errors in the model are normally distributed and coefficients in the model are significant. The residual plots obtained for density are shown in Figure 6. From Figure 6, it was observed that the residuals fall near the straight line, which implies that the developed model coefficients are significant.

**Figure 7:**
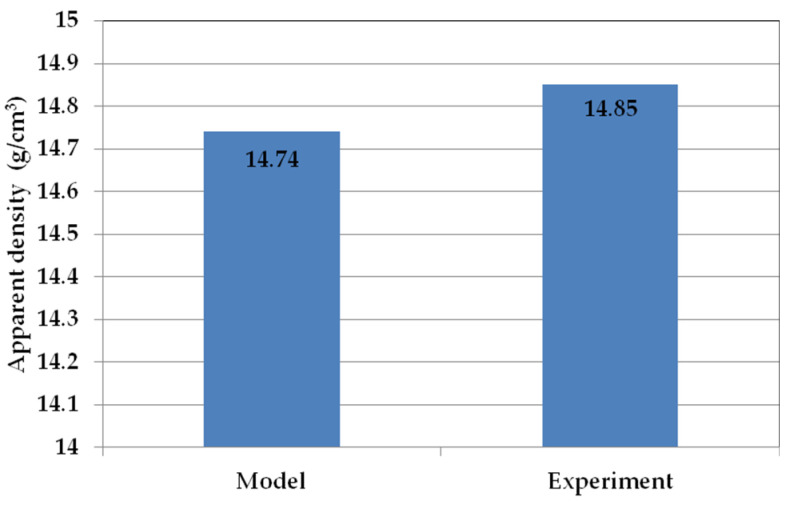
Validation of the developed mathematical model describing the density of the WC-Co composite.

To validate the mathematical model developed to predict apparent density, a confirmatory test was conducted. Spark plasma sintering (SPS) was used to produce the WC-Co composite, with the following parameters: sintering temperature of 1380 °C, heating rate of 100 °C/min and sintering time of 150 s, at a pressing pressure of 45 MPa. The powder mixture preparation process and density measurements were performed according to the methodology described earlier in this paper. Figure 7 shows the model results and experimental verification.

**Figure 8:**
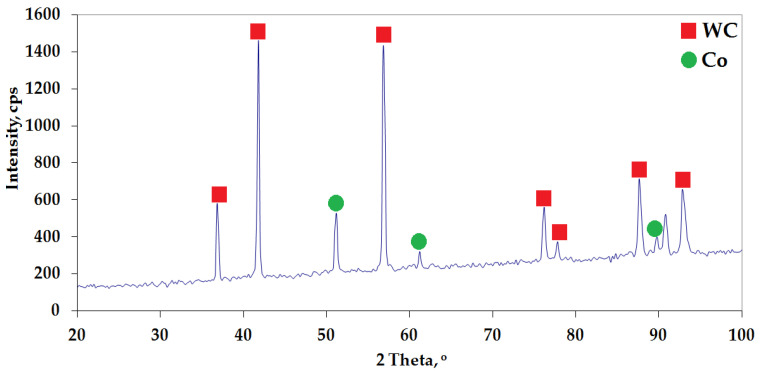
X-ray diffraction of WC-6Co sintered, produced at 1380 °C using the Spark Plasma Sintering (SPS) method.

Based on the results of the mathematical model validation, it was found that the results predicted by the model and the experimental results were consistent within a specific range of parameters. The developed mathematical model predicted the apparent density with an error of only 0.778% compared to the experimental results for a sintering temperature of 1380 °C, a heating rate of 100 °C/min, a sintering time of 150 s and a pressing pressure of 45 MPa. This parameter configuration had not been analysed before. The produced WC-6Co composite sinter with optimal consolidation parameters in the Spark Plasma Sintering (SPS) process achieved a relative density of 97.42%. The developed statistical model will be expanded in the future to include the prediction of sinter hardness. Experiment planning and optimisation is a valuable tool in scientific research. Figure 8 shows the phase composition analysis performed by X-ray diffraction using cobalt lamp radiation with an anode.

The phase composition analysis revealed the presence of tungsten carbide and cobalt in the structure–two phases that are part of the powder mixtures used to produce the sintered material. Based on the obtained diffractogram, no new phases with carbon deficiency (W_2_C) or η phase (W_3_Co_3_C or W_6_Co_6_C) were detected, which could have been formed as a result of the reaction between these two phases. These undesirable phases are characterised by poor wettability by WC and often cause deterioration of the properties of sintered carbides [35]. Table 5 shows the results of WC-6Co sintering tests obtained by validating the statistical model.

A contour plot analysis was performed using statistical modelling. This analysis aimed to examine the relationship between the response variable and the two control variables, illustrating the contours of the predicted response variables.

**Figure 9:**
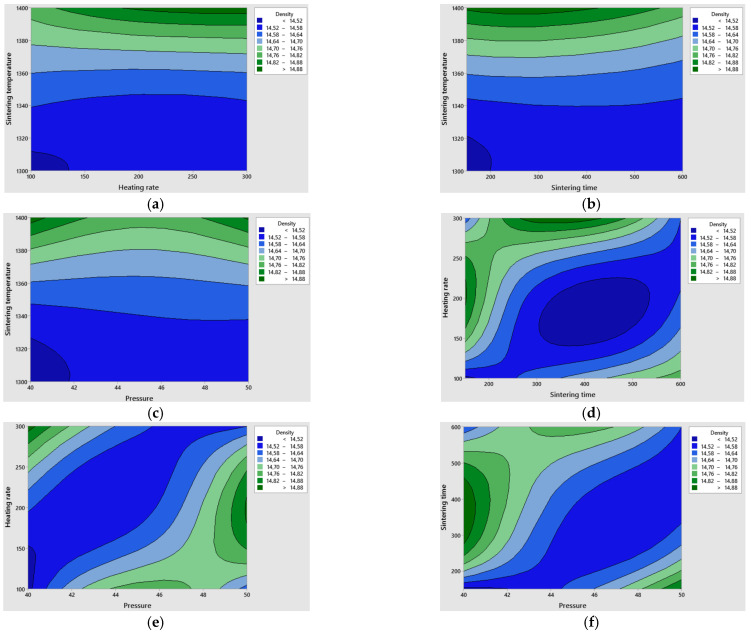
Contour plot for density: (**a**) sintering temperature vs. heating rate, (**b**) sintering temperature vs. sintering time, (**c**) sintering temperature vs. pressure, (**d**) heating rate vs. sintering time, (**e**) heating rate vs. pressure, (**f**) sintering time vs. pressure.

Figure 9 shows contour plots of the relationship between sintering process parameters and sinter apparent density.

Based on the statistical model developed to predict the apparent density of the WC-6Co composite, a design space was defined and represented by the green area in Figure 9a–f. Applying parameters whose intersection points lie within this area guarantees the production of sinters that meet quality requirements, characterised by an apparent density close to the theoretical density, low porosity and excellent mechanical properties.

Within the tested Spark Plasma Sintering process parameters, an apparent density of 14.88 g/cm^3^ is predicted at a sintering temperature of 1370–1400 °C, with a heating rate of 160–250 °C/min, a sintering time of 180–400 s and a pressing pressure of 40–41 MPa.

## 4. Conclusions

The effectiveness of experimental research depends largely on the ability to utilise and apply modern research planning methods, which significantly reduce the number of experiments and also enable the acquisition of valuable and reliable information regarding the research subject.

As a result of the research conducted using DOE methodology, a statistical model was developed demonstrating the influence of parameters on the sintering process of WC-6Co composites using Spark Plasma Sintering technology. Based on this model, a design space for the WC-6Co sinters production process was developed for four dependent variables: sintering temperature, heating rate, sintering time, and pressing pressure. The acquired knowledge will be used to improve the productivity of sinters from the WC-6Co tool materials group, reduce manufacturing costs, and have a positive impact on the environment. The presented research is an example of a practical application of optimisation using the Taguchi method. The following conclusions were drawn based on the research conducted:

1. The optimal combination of spark plasma sintering (SPS) conditions for the WC-6Co composite to achieve a high apparent density was found to be A3-B3-C2-D3.
2. The ANOVA analysis shows that the apparent density of the sintered material was significantly influenced by sintering temperature (93.85%), heating rate (3.38%), pressing pressure (1.69%) and sintering time (1.08%).
3. The statistical model developed demonstrated agreement between the predicted and experimental results. The difference between the two sets of results was 0.741%.
4. The hardness of the sintered material after optimisation was 1809 HV, and its resistance to brittle fracture was 9.48 MPa×m^1/2^.

## Figures and Tables

**Table 1:** Factors and level for sintering experiments.

Factor ^1^	Process Parameters	Units	Level 1	Level 2	Level 3
A	Sintering temperature	°C	1300	1350	1400
B	Heating rate	°C/min^−1^	100	200	300
C	Sintering time	s	150	300	600
D	Pressure	MPa	40	45	50

^1^ A—sintering temperature, B—heating rate, C—sintering time, D—pressure.

**Table 2:** Experimental plan, experimental results and their calculated S/N ratios.

No. Exp.	Controllable Process Parameters				Experimental Results			S/N Rations of Results
	Sintering Temperature (A)	Heating Rate (B)	Sintering Time (C)	Pressure (D)	Density ρ (g/cm^3^)			ρ
					Average ρ	Standard Deviation	Standard Error	
1	1	1	1	1	14.501	0.02645	0.00344	23.227
2	1	2	2	2	14.553	0.03605	0.00479	23.257
3	1	3	3	3	14.58	0.03605	0.00478	23.275
4	2	1	2	3	14.61	0.00577	0.00068	23.293
5	2	2	3	1	14.59	0.02886	0.00338	23.281
6	2	3	1	2	14.60	0.02000	0.00269	23.287
7	3	1	3	2	14.79	0.01154	0.00134	23.399
8	3	2	1	3	14.89	0.01527	0.00200	23.458
9	3	3	2	1	14.91	0.02000	0.00266	23.469

**Table 3:** Mean S/N ratio response table for density.

Symbol	Process Parameters	Mean S/N Ratio				
		Level 1	Level 2	Level 3	Max-Min	Rank
A	Sintering temperature	23.25	23.29	23.44	0.19	1
B	Heating rate	23.31	23.33	23.34	0.04	2
C	Sintering time	23.32	23.34	23.32	0.02	4
D	Pressure	23.33	23.31	23.34	0.03	3

**Table 4:** Variance ANOVA.

Symbol	Process Parameters	Degree of Freedom	Sum of Squares	Mean Squares	% Contribution
A	Sintering temperature	2	0.0610	0.0305	93.85
B	Heating rate	2	0.0022	0.0011	3.38
C	Sintering time	2	0.0007	0.0004	1.08
D	Pressure	2	0.0011	0.0006	1.69
	Total	8	0.0650		100.00

**Table 5:** Test results following the optimisation of the parameters for the Spark Plasma Sintering process of the WC-6Co composite.

Sample	Porosity (%)			Hardness	Resistance to Brittle Fracture
	P_o_	P_z_	P_c_	HV30	K_IC_
WC-6Co	1.635	0.948	2.583	1809	9.48

P_o_—open porosity, P_z_—closed porosity, P_c_—total porosity.

## Data Availability

The original contributions presented in this study are included in the article. Further inquiries can be directed to the corresponding authors.
